# Comparison of Two Bayesian Methods in Evaluation of the Absence of the Gold Standard Diagnostic Tests

**DOI:** 10.1155/2019/1374748

**Published:** 2019-08-21

**Authors:** Taishun Li, Pei Liu

**Affiliations:** Department of Epidemiology and Biostatistics, School of Public Health, Southeast University, 87 Ding Jiaqiao Rd., Nanjing 210009, China

## Abstract

**Objective:**

The Bayesian model plays an important role in diagnostic test evaluation in the absence of the gold standard, which used the external prior distribution of a parameter combined with sample data to yield the posterior distribution of the test characteristics. However, the correlation between diagnostic tests has always been a problem that cannot be ignored in the Bayesian model evaluation. This study will discuss how different Bayesian model, correlation scenarios, and prior distribution affect the outcome.

**Methods:**

The data analyzed in this study was gathered during studies of patients presenting to the Nanjing Chest Hospital with suspected tuberculosis. The diagnostic character of T-SPOT.Tb and KD38 tuberculosis antibody test were evaluated in different Bayesian model, and discharge diagnosis as a gold standard was used to verify the model results in the end.

**Result:**

The comparison of four models under the conditional independence situation found that Bayesian probabilistic constraint model was consistent with the Conditional Covariance Bayesian model. The results were mainly affected by prior information. The sensitivity and specificity of the two tests in Conditional Covariance Bayesian model in prior constraint situation were considerably higher than the Bayesian probabilistic constraint model in prior constraint situation. The results of the four models under the conditional dependence situation were similar to the conditional independence situation; p_D_ was also negative with no prior constraint situation in both model Bayesian probabilistic constraint model and Conditional Covariance Bayesian model. The Deviance Information Criterion of Bayesian probabilistic constraint model was close to model Conditional Covariance Bayesian model, but p_D_ of Conditional Covariance Bayesian model in Prior constraint situation (p_D_=2.40) was higher than the Bayesian probabilistic constraint model in Prior constraint situation (p_D_=1.66).

**Conclusion:**

The result of Conditional Covariance Bayesian model in prior constraint with conditional independence situation was closest to the result of gold standard evaluation in our data. Both of the two Bayesian methods are the feasible way for the evaluation of diagnostic test in the absence of the gold standard diagnostic. Prior source, priority number, and conditional dependencies should be considered in the method selection, the accuracy of posterior estimation mainly depending on the prior distribution.

## 1. Introduction

Sensitivity and specificity as the reference value of the ability to detect sick and healthy patients are used in diagnostic test evaluation with a gold standard test. However, in clinical practice, the gold standard tests are not given in patients due to expensive or invasive reasons [1]. The absence of a gold standard is a common problem in clinical practice and diagnostic research studies.

Some studies try to evaluate the diagnostic test characteristics by combining multiple diagnostic tests in the absence of a gold standard [2, 3]. Due to the fact that the sensitivity and specificity of diagnostic tests in the estimation process are unknown variables, the biggest difficulty is that the number of parameters of estimation exceeds the number of degrees of freedom provided by the data. For example, when two nongold standard diagnostic tests are used, only three degrees of freedom are provided, but the sensitivity and specificity of the two tests and the prevalence of the disease need to be estimated for at least five unknown parameters; if the correlation between the two tests is considered, there are more parameters to be estimated.

In classical statistical view, sensitivity and specificity are regarded as fixed parameters and the population prevalence is calculated from them. However, it has been proved that sensitivity and specificity are not fixed values, but change with external factors [4, 5]. The sensitivity and specificity of diagnostic tests in the estimation process are unknown, and their values are often independent of the sample data [6]. According to the Bayesian view, any unknown parameter can be regarded as a random variable, and its unknown state can be described by a probability distribution. This probability distribution is called a prior distribution; the prior constraints on Bayesian methods can compensate for the lack of freedom. Of course, prior information needs to be specified by external data, which can be the expert opinion or historical research.

Bayesian methods have been increasingly used to evaluate the true accuracy of diagnostic tests in the absence of a gold standard [7–9] for two reasons. On the one hand, prior information in the Bayesian framework about the sensitivities and specificities of the tests can be obtained from experimental results or other studies; if there is no data source, it can be replaced by an expert prior [10]. The Bayesian analysis allows us to combine external prior information with the data likelihood to yield the posterior estimation of unknown parameters such as the prevalence and diagnostic test characteristics [11]. On the other hand, with the development of computer technology and professional Bayesian analysis software such as OpenBUGS, the computational problems in the Bayesian method have been solved by the efficient Markov chain Monte Carlo (MCMC) algorithms for sampling and summarizing posterior distributions [10].

In the combined application of multiple diagnostic tests, the interdependence between different tests also needs to be considered in the Bayesian model. If two tests have the same biological attribute, it is logical to believe that the tests are conditionally dependent; if the result is positive in one test, the result of another test is likely to be positive [12]. Several approaches try to take conditional interdependence of different tests into account in Bayesian models. One method is to calculate the correlation coefficient directly and incorporate the covariance into the Bayesian model [13]. The other method is to use probabilistic constraints to transform the interdependence of different tests into conditional probabilities and then to construct a Bayesian model based on conditional probabilities [14]. Both approaches will have their application scenarios.

Under the basic Bayesian framework, the Bayesian method is very flexible when considering various influencing factors. The correlation scenarios, prior distribution, and the number of the prior parameters are the important factors that cannot be ignored in the Bayesian estimation. The objective of this study is to compare the two Bayesian methods under different scenarios with tuberculosis (TB) data and to explore the application scenarios for each of the two Bayesian methods.

## 2. Methods

### 2.1. Study Patients and Diagnostic Tests

The data analyzed in this study was gathered during studies of patients presenting to the Nanjing Chest Hospital with suspected tuberculosis. In brief, a case report of patients was collected between June and October 2015 at the Nanjing Chest Hospital. Informed consent was completed for all participated in the study. T-SPOT.Tb and KD38 tuberculosis antibody test was combined as the nongold standard diagnostic test to estimate the prevalence and diagnostic test characteristic. The discharge diagnosis was used to verify the model results.

### 2.2. Conditional Covariance Bayesian Model

#### 2.2.1. The Description of Conditional Covariance Bayesian Model

Conditional covariance Bayesian model directly considers the correlation between the two tests and estimates the conditional correlation between two diagnostic tests using the covariance between tests within the diseased and nondiseased populations. Two diagnostic test evaluation models without a gold standard were shown in Table 1, and parameter explanation was listed as follows: (1) *a*_*ij*_: i=0,1 represents the negative and positive results of the Test1, respectively, and j=0,1 represents the negative and positive results of the Test2, correspondingly; (2) t represents the number of real patients; (3) n represents the total number; (4) *t*_*ij*_: *t*_*ij*_ is the positive potential true value corresponding to *a*_*ij*_; and (5) *a*_*ij*_ − *t*_*ij*_: *a*_*ij*_ − *t*_*ij*_ is the negative potential true value. Potential true values are those that cannot be observed directly but are close to the gold standard under certain conditions. In the Conditional Covariance Bayesian model, the conditional correlation between the two diagnostic tests was estimated by calculating the covariance between tests within each disease class [12, 15]. Bayesian conditional covariance models use the method of Nandini Dendukuri et al. and the description of the methods partly reproduces their wording [13]. Model construction in both independent and dependent scenarios was provided as supporting information (Text S1).

#### 2.2.2. Likelihood Function of the Conditional Covariance Bayesian Model

Vector A = (*a*_11_, *a*_10_, *a*_01_, *a*_00_) represents the actual result of two diagnostic tests (1 is positive, 0 is negative); the probability value P corresponding to vector a is equal to (*P*_11_, *P*_10_, *P*_01_, *P*_00_). The sensitivity of the first and second tests is *se*_1_ and *se*_2_, the specificity of the two tests is *sp*_1_ and *sp*_2_.

*(a) The Covariance under Positive Conditions of the True Result*

(1) $$\begin{array}{c} cov\left(\;D_{p}\;\right)={se}_{11}-{se}_{1}{se}_{2}. \end{array}$$

*(b) The Covariance under Negative Conditions of the True Result*

(2) covDn=sp11−sp1sp2.se11=PT1+,T2+ ∣ D+,sp11=PT1−,T2− ∣ D−.

*(c) The Correlation Coefficient under the True Disease Condition is Positive ρ*_*D*+_

(3) $$\begin{array}{c} \rho_{D+}=\frac{cov\left(D_{p}\right)}{\sqrt{{Se}_{1}\left(1-{Se}_{1}\right){Se}_{2}\left(1-{Se}_{2}\right)}} \end{array}$$

*(d) The Correlation Coefficient under the True Disease Condition is Negativeρ*_*D*−_

(4) $$\begin{array}{c} \rho_{D-}=\frac{cov\left(D_{n}\right)}{\sqrt{{Sp}_{1}\left(1-{Sp}_{1}\right){Sp}_{2}\left(1-{Sp}_{2}\right)}} \end{array}$$

*(e) Likelihood Function*. The likelihood function of the Conditional Covariance Bayesian model is a multinomial likelihood function.

(5) L=Pa11,a10,a01,a00 ∣ p,se1,se2,sp1,sp2,covDp,covDn,t11,t10,t01,t00∝pse1se2+covDpt11∗pse11−se2−covDpt10∗pse21−se1−covDpt01∗p1−se21−se1+covDpt00∗1−p1−sp21−sp1+covDna11−t11∗1−p1−sp1sp2−covDna10−t10∗1−p1−sp2sp1−covDna01−t01∗1−psp2sp1+covDna00−t00

#### 2.2.3. Prior Information about the Conditional Covariance Bayesian Method

According to the Bayesian principle, the conjugate distribution of the binomial distribution is the beta distribution. The prevalence, sensitivities, and specificities are assumed to follow beta prior distribution, list like the following:

(6) Se1~betaa1,b1,Se2~betaa2,b2Sp1~betaa3,b3,Sp2~betaa4,b4p~betaa5,b5

The prior information of the above parameters (*Se*_1_, *Se*_2_, *Sp*_1_, *Sp*_2_, *p*) was gathered from the previous study in China. For example, the prior information of sensitivity and specificity for the T-SPOT method was gathered from 18 previous similar published researches in a different area of China. According to the data (mean, standard deviation) calculated from the historical prior, the parameters (a and b) of the prior beta distribution of unknown variables are obtained (Table 2).

In practical application, there is a lack of available prior information in covariance cov(*D*_*p*_) and cov(*D*_*n*_); the covariance is random variables varying in a finite range as follows:

(7) Se1−11−Se2covDp≤min⁡Se1,Se2−Se1Se2Sp1−11−Sp2covDn≤min⁡Sp1,Sp2−Sp1Sp2

Since the positive correlation between the two tests is the actual consideration, but the lower limit value in the above expression is always negative, the lower limit value was artificially fixed to zero. Only when the distribution is uniform, the entropy can reach the maximum value. So the prior distribution is defined as follows:

(8) covDp~Uniform0,min⁡Se1,Se2−Se1Se2,covDn~Uniform0,min⁡Sp1,Sp2−Sp1Sp2.

### 2.3. Bayesian Probabilistic Constraint Model

#### 2.3.1. The Description of Bayesian Probabilistic Constraint Model

The conditionally independent assumption is usually made when two diagnostic tests were combined. However, the conditionally independent assumption cannot easily be made when the two diagnostics have a similar biologic mechanism; extra information will be required in the estimation process [16]. When the number of estimable parameters exceeds the number of parameters to estimate, the Bayesian probabilistic constraint model is to add the constraint on the parameters. These constraints usually come from external information, such as historical study or expert opinion. In the Bayesian method, we call it prior information, and it is also the explanation for the constraint of the Bayesian probabilistic constraint model.

However, in some cases, it is often difficult to directly specify external prior information for some parameters, such as the covariance in the Conditional Covariance Bayesian model. The prior distributions for the covariance are quite difficult to elicit from experts or other studies, because they are not the indicator used in a real-life situation. In Bayesian probabilistic constraint model, prior information on conditional probabilities is easier to specify [14]. Therefore, in the Bayesian probabilistic constraint model, the correlation coefficients between the two diagnostic tests are not calculated directly, some restriction will be imposed on the parameter estimates. We just elicited the information from experts on the conditional performance on one test given the results of another test. The Bayesian probabilistic constraint model uses the method of Berkvens, D. et al. and the description of the method partly reproduces their wording [14]. Model construction in both independent and correlated scenarios was provided as supporting information (Text S2).

#### 2.3.2. Likelihood Function of the Bayesian Probabilistic Constraint Model

The likelihood function was used to express the cell probabilities of the collapsed 2^*h*+1^, the table in terms of the prevalence of the disease, *D*^+^(*D*^−^) indicated that the subject was (was not) diseased; *T*^+^(*T*^−^) indicated a positive (negative) result with test T. *i*_*t*_ also indicated the test condition (0 indicated a negative result, and 1 indicated the positive result). An example was listed as follows:

(9) T10∩T20=P00=PD+(1−PT1+ ∣ D+(1−PT2+ ∣ D+∩T1−+1−PD+PT1− ∣ D−PT2− ∣ D−∩T1−=θ11−θ21−θ5+1−θ1θ3θ6

*Likelihood function listed as follows:*

(10) PT1i1∩⋯∩Thih=PD+·∏t=1h1−it−−1itPTt+ ∣ D+⋂t′ ∣ t>1t−1Tt′it′+1−PD+·∏t=1hit+−1itPTt− ∣ D−⋂t′ ∣ t>1t−1Tt′it′

#### 2.3.3. Prior Information about the Bayesian Probabilistic Constraint Model

The prior information about Bayesian probabilistic constraints model was collected from four experienced tuberculosis physicians. The tuberculosis physician answered the probability of the parameter under the defined question. After obtaining the expert answer, the mean of each parameter had been calculated, and the prior distribution also had been specified. This study was a joint evaluation of two diagnostic tests, *θ*_1_ - *θ*_7_ were used for conditional probabilities, and the meaning of the specific reference to each conditional probability was found in S2. In this article, we assumed that the prior distribution of each conditional probability obeyed the beta distribution with two parameters (alpha and beta); specific information was listed in Table 3.

### 2.4. Model Evaluation and Verification

All parameters in two Bayesian methods were estimated with 95% credible intervals using OpenBUGS 3.2.3[17]. The OpenBUGS code of this study was provided as an attachment file (Text S3). Deviance information criteria (DIC) were used to evaluate the models fit and to verify whether the prior information is against data results [18]. During the model building process, the DIC was minimized, it aims to find the simplest and best-fit model, and the lower the DIC value, the simpler and fitter the model [19]. The number of parameters (*p*_*D*_) also represented the complexity of the model and indicated the final reduction in the number of parameters needs to be estimated.

The prediction accuracy of the different models was evaluated using clinical discharge diagnosis as the gold standard. The clinical discharge diagnosis was a comprehensive judgment made by doctors according to various diagnostic tests, expert experience, and disease progression.

## 3. Results

In total, 637 patients with suspected tuberculosis were included in the study. The mean age was 50.12 years (range 15-90 years); 61.3% of the patients were male and 38.7% were female. 130 patients (20.41%) were negative for T-SPOT.TB test and KD38 tuberculosis antibody test, 235 patients (36.89%) were positive for both of them, the four possible combinations of results for the two tests were listed (Table 4).

### 3.1. Conditional Independence Situation

Four models under conditional independence situation were applied to the data for the two tests, which assumed that the result of the first test had no influence on the result of the second test. Using the observed of the two tests as the sample data, combined with prior information, we calculated the posterior distribution of sensitivity and specificity of the two tests (Table 5). Under the premise of the conditional independence situation, the Bayesian probabilistic constraint model was consistent with the Conditional Covariance Bayesian model. Therefore, the results of the two models were the same with no prior constraint. Under the condition of prior situation, the results were affected by prior information. The sensitivity and specificity of the two tests in model PC were considerably higher than those predicted in the model PP. The tuberculosis prevalence was estimated to be 63.6% (95% credible interval 43.5%-77.3%) in model PC, being considerably higher than model PP (53.4%, 95% credible interval 45.2 %-61.4%). DIC and *p*_*D*_ of the different model under conditional independence situation were compared (Table 6). *p*_*D*_ was negative with no prior constraints in both NP and NC models, which indicated all our parameters were estimable. The model PC reduced the number of parameters (*p*_*D*_) which was 2.26 and had smaller DIC than the model PP.

### 3.2. Conditional Dependence Situation

Conditional dependence situation assumed that the two diagnostic tests could be correlated. The posterior distributions of sensitivity and specificity of the two tests under conditional dependence situation were evaluated by four models (Table 7). Whether or not there was a prior constraint, the posterior estimation results of five parameters in the Conditional Covariance Bayesian models were higher than Bayesian probabilistic constraint model. The result of the four models under conditional dependence situation was similar to the conditional independence situation, especially in the case of models with prior constraint. The DIC of the different model under conditional dependence were compared (Table 8), which were also negative with no prior constraints in both model NP and NT. The DIC of model PP were close to model PC, but the *p*_*D*_ of the model PC (*p*_*D*_=2.40) was higher than model PP (*p*_*D*_=1.66).

### 3.3. Impact of Prior Number

The Conditional Covariance Bayesian model was chosen to explore the influence of the prior number on the posterior estimation because it has only five unknown parameters corresponding to only five prior distributions, which was convenient for simulation studies. When the number of priors was equal to n, it means that the rest of the prior (5-n) was prior without information. From the results of simulation under conditional independence, when the prior number was three, the estimation result and the model were stable (Table 9), which were very close to the full prior estimation results. Similarly, models and the results were stable when the number of prior information was three in conditional dependence situation (Table 10).

### 3.4. Model Validation

The patient discharge diagnosis was used as the gold standard to evaluate the sensitivity and specificity of two diagnostic tests (Table 11), tuberculosis prevalence in our population was estimated to be 82.9% (95% confidence interval 79.7%-85.6%), the sensitivity and specificity of T-SPOT.TB test were 0.739 and 0.670, and the sensitivity and specificity of KD38 tuberculosis antibody test were 0.549 and 0.761. The result of the model PC in conditional independence situation was closest to the result of the gold standard evaluation.

## 4. Discussion

With the development of computer technology and Bayesian theory, Bayesian model has been widely used in the practice of medical research. In the evaluation of diagnostic tests, when the real disease status is unknown and there is no gold standard, Bayesian method can be used to integrate external prior information and sample data to evaluate diagnostic test characteristics by combining two or more imperfect tests [20, 21]. However, due to the flexibility of the Bayesian model, its estimated results are affected by many factors. The consideration of correlation and the choice of prior distribution are the most important influencing factors for the posterior estimation.

The result of the different model indicated that the estimate of prevalence rate and diagnostic test characteristics depends on the model chosen, prior selection, and dependencies between tests. Compared with the gold standard verification, the result of model PC in conditional independence situation was closest to the result of the gold standard evaluation. The reasons for the above phenomenon may be as follows: firstly, the weak correlation between diagnostic tests cannot have a significant effect on the result; secondly, the prior constraint model can reflect the real situation of the diagnostic test than the nonpriority constraint model; thirdly, the objective prior information from previous studies is more accurate than expert opinion.

The dependencies between diagnostic tests have always been a key issue for Bayesian models. The results of the two Bayesian models showed that the change for the possibility of conditional dependence between diagnostic tests had a certain impact on the posterior estimates of diagnostic test characteristics. The two Bayesian methods deal with the conditional dependencies between tests in different ways. Conditional covariance Bayesian method combined prior information on covariance parameters with the test result to calculate the posterior distribution of the correlation coefficients. However, obtaining the prior distribution of covariance from experts or literature is pretty hard, because it is not specific parameters in a real-life situation. In addition, the complex correlation will be difficult to estimate with multiple diagnostic tests. In order to overcome this problem, the Bayesian probabilistic constraint model does not directly calculate the correlation coefficient, it just elicits prior information for experts on the conditional performance on one test given the results of another test, and this can be easier to answer by experts in a real-life situation. However, such prior information from this model is the expert subjective opinion, and its credibility is not better than objective prior information.

Our results showed that the likelihood functions of the two Bayesian methods were consistent with the conditions of independence situation, and the posterior estimation strongly depended on the prior information. The results of the two Bayesian methods both illustrated that posterior estimation was mainly affected by the available prior information. Hence, it is very important to elicit the prior distribution accurately. On the one hand, the objectivity of prior information is crucial. In the Conditional Covariance Bayesian method, the prior distribution of unknown parameters can be gathered from previous studies, and objective prior information is suggested to ensure the credibility of the result. In the Bayesian probabilistic constraint model, it may be easier to specify expert prior information for unknown parameters, but it is also significant to realize that the unstable expert opinion may have a great impact on the result; if you use the prior by different experts, you may end up with distinctive conclusions. On the other hand, the number of prior information as well has an important effect on the stability of the results. As we all know, the more the number of a prior, the more accurate the result, but it will increase the burden of obtaining prior information. Our results show that three prior distributions can achieve full prior results in the Conditional Covariance Bayesian method. Therefore, obtaining stable results based on minimal prior information is the best choice.

In fact, the influences of prior information and dependencies on the results are inseparable. Because the correlation coefficient itself is an unknown parameter, it also requires the prior distribution. In the evaluation of diagnostic tests in the absence of the gold standard, many factors should be considered in the method selection. DIC is also an important index of the model selection. Both the Conditional Covariance Bayesian method and the Bayesian probabilistic constraint method have their specific applicable scenarios; the users should choose the appropriate method according to the needs of the actual situation. When there are only two diagnostic tests and the correlation coefficient can be objectively specified, the Conditional Covariance Bayesian method is more applicable. The Conditional Covariance Bayesian method could also be extended to include more than two tests by adding more covariance in the model. At this time, the calculation of covariance will become complex, and the determination of prior distribution will be more difficult. Hence, from the point of view of practical application, the Bayesian probabilistic constraint method is more suitable when there are more than two combined diagnostic tests without gold standards. Finally, although these two methods are not perfect, they provide a feasible way for the evaluation of diagnostic test in the absence of a gold standard diagnostic; at the same time, it is of great significance to promote the application of Bayesian method in medical research.

## 5. Conclusion

Both of the two Bayesian methods are the feasible way for the evaluation of diagnostic test in the absence of a gold standard diagnostic. Prior source, priority number, and conditional dependencies should be considered in the method selection, the accuracy of posterior estimation mainly depending on the prior distribution.

## Figures and Tables

**Table 1:** Two diagnostic test evaluation models without the gold standard.

		True result(+)		Total	True result(-)		Total
		Test2			Test2		
		+	-		+	-	
Test1	+	*t*_11_	*t*_10_	*t*_−_{1.}	*a*_11_ − *t*_11_	*a*_10_ − *t*_10_	*a*_−_{1.} − *t*_−_{1.}
	-	*t*_01_	*t*_00_	*t*_−_{0.}	*a*_01_ − *t*_01_	*a*_00_ − *t*_00_	*t*_−_{1.} − *t*_−_{0.}
Total		*t*_−_{.1}	*t*_−_{.0}	t	*a*_−_{.1} − *t*_−_{.1}	*a*_−_{.0} − *t*_−_{.0}	n − t

**Table 2:** The prior information of the Conditional Covariance Bayesian model.

Method	Knot	N	Mean	VAR	SD	CI	a	b
T-SPOT	*Se*_1_	18	0.893	0.002	0.049	0.869-0.917	41.770	5.005
	*Sp*_1_	18	0.848	0.010	0.098	0.799-0.896	10.082	1.807
KD38	*Se*_2_	9	0.572	0.018	0.132	0.47-0.674	7.207	5.393
	*Sp*_2_	9	0.717	0.007	0.082	0.654-0.781	20.067	7.920
Prev	p	11	0.417	0.023	0.153	0.315-0.52	3.991	5.579

*Se*_1_: sensitivity of T-SPOT; *Sp*_1_: specificity of T-SPOT; *Se*_2_: sensitivity of KD38; *Sp*_2_: specificity of KD38; p: prevalence within the patients in the study; N: the number of published research; VAR: variance; SD: standard deviation; CI: credible interval; a and b were the parameters of the prior distribution.

**Table 3:** The prior information about the Bayesian probabilistic constraint model.

Parameter	Knot	N	D1	D2	D3	D4	Mean	Alpha	Beta
*θ*_1_	P2.5	4	0.3	0.5	0.7	0.3	0.450	75.83	66.73
	P97.5	4	0.55	0.7	0.8	0.4	0.613		
*θ*_2_	P2.5	4	0.9	0.7	0.8	0.6	0.572	70.84	35.86
	P97.5	4	0.99	0.8	0.9	0.8	0.750		
*θ*_3_	P2.5	4	0.7	0.7	0.7	0.8	0.417	21.90	16.21
	P97.5	4	0.8	0.8	0.8	0.9	0.725		
*θ*_4_	P2.5	4	0.7	0.4	0.4	0.75	0.563	99.74	56.18
	P97.5	4	0.8	0.6	0.6	0.85	0.713		
*θ*_5_	P2.5	4	0.3	0.3	0.4	0.1	0.275	53.43	100.05
	P97.5	4	0.5	0.5	0.5	0.2	0.425		
*θ*_6_	P2.5	4	0.7	0.4	0.4	0.75	0.563	143.25	85.38
	P97.5	4	0.8	0.6	0.5	0.85	0.688		
*θ*_7_	P2.5	4	0.2	0.3	0.6	0.5	0.400	112.32	130.69
	P97.5	4	0.3	0.5	0.7	0.6	0.525		

D1: Doctor 1; D2: Doctor 2; D3: Doctor 3; D4: Doctor4. Alpha and Beta are two parameters of the beta distribution.

**Table 4:** The results of 637 persons subjected to 2 diagnostic tests.

T-SPOT	KD38	Count
-	-	130
-	+	81
+	-	191
+	+	235

- indicates negative test result and + positive test result.

**Table 5:** The posterior estimation of four models using the TB data under conditional independence situation.

Situation	Method	Model	Knot	Mean	SD	Median	95% Bayesian CI
							(P2.5-P97.5)
No prior constraints	Bayesian probabilistic constraint model	NP	*Se*_1_	0.660	0.243	0.762	0.503-0.841
			*Sp*_1_	0.418	0.251	0.426	0.203-0.584
			*Se*_2_	0.511	0.251	0.568	0.302-0.688
			*Sp*_2_	0.569	0.249	0.631	0.377-0.759
			p	0.512	0.194	0.515	0.361-0.667
	Conditional Covariance Bayesian model	NC	*Se*_1_	0.618	0.250	0.631	0.071-0.974
			*Sp*_1_	0.377	0.250	0.295	0.025-0.929
			*Se*_2_	0.468	0.255	0.435	0.036-0.947
			*Sp*_2_	0.526	0.253	0.480	0.055-0.962
			p	0.498	0.194	0.497	0.148-0.850

Prior constraints	Bayesian probabilistic constraint model	PP	*Se*_1_	0.738	0.036	0.739	0.714-0.762
			*Sp*_1_	0.454	0.051	0.453	0.419-0.487
			*Se*_2_	0.585	0.031	0.585	0.563-0.606
			*Sp*_2_	0.525	0.028	0.525	0.506-0.544
			p	0.534	0.042	0.534	0.506-0.562
	Conditional Covariance Bayesian model	PC	*Se*_1_	0.898	0.039	0.910	0.814-0.963
			*Sp*_1_	0.765	0.125	0.775	0.515-0.968
			*Se*_2_	0.594	0.048	0.586	0.523-0.712
			*Sp*_2_	0.679	0.048	0.676	0.592-0.780
			p	0.636	0.086	0.650	0.435-0.773

*Se*_1_: sensitivity of T-SPOT; *Sp*_1_: specificity of T-SPOT; *Se*_2_: sensitivity of KD38; *Sp*_2_: specificity of KD38;  p: prevalence within the patients in the study; SD: standard deviation; CI: confidence interval.

**Table 6:** The results of fitting indicator between four models under the conditional independence situation.

Model	DIC	*p*_*D*_	p	T-SPOT		KD38	
				Se	Sp	Se	Sp
Method NP	3.128	-19.05	0.512	0.660	0.418	0.511	0.569
Method NC	1.404	-20.80	0.498	0.660	0.418	0.511	0.569
Method PP	38.56	1.343	0.5334	0.738	0.454	0.585	0.525
Method PC	24.15	2.264	0.636	0.898	0.765	0.594	0.679

Se: sensitivity; Sp: specificity;  p: prevalence within the patients in the study.

**Table 7:** The posterior estimation of four models using the TB data under conditional dependence situation.

Situation	Method	Model	Knot	Mean	SD	Median	95% Bayesian CI
							(P25-P75)
No prior constraints	Bayesian probabilistic constraint model	NP	*Se*_1_	0.626	0.218	0.665	0.516-0.773
			*Sp*_1_	0.369	0.217	0.330	0.222-0.476
			*Se*_2_	0.490	0.167	0.495	0.391-0.585
			*Sp*_2_	0.508	0.167	0.503	0.414-0.616
			p	0.498	0.248	0.497	0.305-0.692
	Conditional Covariance Bayesian model	NC	*Se*_1_	0.690	0.183	0.708	0.622-0.807
			*Sp*_1_	0.435	0.226	0.390	0.287-0.568
			*Se*_2_	0.547	0.197	0.535	0.455-0.657
			*Sp*_2_	0.571	0.213	0.562	0.444-0.722
			p	0.553	0.254	0.578	0.366-0.755

Prior constraints	Bayesian probabilistic constraint model	PP	*Se*_1_	0.713	0.036	0.714	0.689-0.738
			*Sp*_1_	0.423	0.051	0.421	0.387-0.456
			*Se*_2_	0.535	0.025	0.535	0.518-0.552
			*Sp*_2_	0.537	0.022	0.537	0.522-0.552
			p	0.538	0.042	0.539	0.510-0.567
	Conditional Covariance Bayesian model	PC	*Se*_1_	0.904	0.037	0.907	0.881-0.931
			*Sp*_1_	0.796	0.119	0.814	0.715-0.892
			*Se*_2_	0.588	0.055	0.580	0.551-0.614
			*Sp*_2_	0.677	0.068	0.677	0.631-0.723
			p	0.649	0.076	0.662	0.608-0.701

*Se*_1_: sensitivity of T-SPOT; *Sp*_1_: specificity of T-SPOT; *Se*_2_: sensitivity of KD38; *Sp*_2_: specificity of KD38;  p: prevalence within the patients in the study; SD: standard deviation; CI: confidence interval.

**Table 8:** The results of fitting indicator between four models under the conditional dependence situation.

Model	DIC	PD	p	T-SPOT		KD38	
				Se	Sp	Se	Sp
Method NP	19.01	-3.005	0.498	0.626	0.369	0.490	0.508
Method NC	14.32	-7.692	0.553	0.690	0.435	0.547	0.571
Method PP	24.26	1.657	0.538	0.713	0.423	0.535	0.537
Method PC	24.40	2.40	0.649	0.904	0.797	0.588	0.677

Se: sensitivity; Sp: specificity;  p: prevalence within the patients in the study.

**Table 9:** The impact of the number of prior information on the assessment result (conditional independence situation).

Number	DIC	*p*_*D*_	p	T-SPOT		KD38	
				Se	Sp	Se	Sp
0	1.404	-20.8	0.5059	0.74	0.4078	0.5533	0.6127
1	18.12	-3.992	0.4524	0.8125	0.4462	0.6763	0.6624
2	24.03	2.0	0.4357	0.8935	0.4948	0.7144	0.6678
3	24.21	2.174	0.6421	0.9056	0.7688	0.5856	0.6617
4	24.5	2.497	0.6482	0.9054	0.7788	0.5827	0.6612

Se: sensitivity; Sp: specificity;  p: prevalence within the patients in the study.

**Table 10:** The impact of the number of prior information on the assessment result (conditional dependence situation).

Number	DIC	*p*_*D*_	p	T-SPOT		KD38	
				Se	Sp	Se	Sp
0	14.32	-7.692	0.5776	0.7081	0.3903	0.5353	0.562
1	20.43	-1.594	0.4503	0.7325	0.3825	0.5814	0.5804
2	24.32	2.298	0.455	0.8941	0.5115	0.5963	0.6073
3	24.56	2.503	0.6526	0.9075	0.7941	0.5548	0.6185
4	24.69	2.652	0.6579	0.9081	0.8063	0.5575	0.6219

Se: sensitivity; Sp: specificity;  p: prevalence within the patients in the study.

**Table 11:** Post-hoc model validation with the gold standard.

Indicator	T-SPOT		KD38	
	No.*∗*	95% CI	No.*∗*	95% CI
Sensitivity	390/528	0.739 (0.699-0.776)	290/528	0.549 (0.507-0.591)
Specificity	73/109	0.670 (0.573-0.757)	83/109	0.761 (0.670-0.838)
Prevalence	528/637	0.829 (0.797-0.857)	528/637	0.829 (0.797-0.857)

*∗* Patient discharge diagnosis was used as the gold standard.

## Data Availability

The data used to support the findings of this study are included within the supplementary information file (Table S1).

## Supplementary Materials

Supplementary file
* Text S1:* two scenarios of Conditional Covariance Bayesian model.

Supplementary file
* Text S2:* two scenarios of the Bayesian probabilistic constraint model.

Supplementary file
* Text S3:* OpenBugs code.

Supplementary file
* Table S1:* origin dataset.

## References

[1] Buzoianu M., Kadane J. B. (2008). Adjusting for verification bias in diagnostic test evaluation: a Bayesian approach. *Statistics in Medicine*.

[2] Perpiñá M., Pellicer C., de Diego A., Compte L., Macián V. (1993). Diagnostic value of the bronchial provocation test with methacholine in asthma. *Chest*.

[3] Schumacher S. G., van Smeden M., Dendukuri N. (2016). Diagnostic test accuracy in childhood pulmonary tuberculosis: a bayesian latent class analysis. *American Journal of Epidemiology*.

[4] Greiner M., Gardner I. A. (2000). Epidemiologic issues in the validation of veterinary diagnostic tests. *Preventive Veterinary Medicine*.

[5] Begg C. B. (1987). Biases in the assessment of diagnostic tests. *Statistics in Medicine*.

[6] Lesaffre E., Speybroeck N., Berkvens D. (2007). Bayes and diagnostic testing. *Veterinary Parasitology*.

[7] Wang C., Turnbull B. W., Nielsen S. S., Grohn Y. T. (2011). Bayesian analysis of longitudinal Johne's disease diagnostic data without a gold standard test. *Journal of Dairy Science*.

[8] Vilar M. J., Ranta J., Virtanen S., Korkeala H. (2015). Bayesian estimation of the true prevalence and of the diagnostic test sensitivity and specificity of enteropathogenic yersinia in finnish pig serum samples. *BioMed Research International*.

[9] Tang Z., Zeng F., Yu X., Zhou L. (2014). Bayesian estimation of cardiovascular autonomic neuropathy diagnostic test based on baroreflex sensitivity in the absence of a gold standard. *International Journal of Cardiology*.

[10] Branscum A. J., Gardner I. A., Johnson W. O. (2005). Estimation of diagnostic-test sensitivity and specificity through Bayesian modeling. *Preventive Veterinary Medicine*.

[11] Dorny P., Phiri I., Vercruysse J. (2004). A Bayesian approach for estimating values for prevalence and diagnostic test characteristics of porcine cysticercosis. *International Journal for Parasitology*.

[12] Gardner I. A., Stryhn H., Lind P., Collins M. T. (2000). Conditional dependence between tests affects the diagnosis and surveillance of animal diseases. *Preventive Veterinary Medicine*.

[13] Dendukuri N., Joseph L. (2001). Bayesian approaches to modeling the conditional dependence between multiple diagnostic tests. *Biometrics*.

[14] Berkvens D., Speybroeck N., Praet N., Adel A., Lesaffre E. (2006). Estimating disease prevalence in a Bayesian framework using probabilistic constraints. *Epidemiology*.

[15] Dendukuri N., Rahme E., Bélisle P., Joseph L. (2004). Bayesian sample size determination for prevalence and diagnostic test studies in the absence of a gold standard test. *Biometrics*.

[16] Tu X. M., Kowalski J., Jia G. (1999). Bayesian analysis of prevalence with covariates using simulation-based techniques: applications to HIV screening. *Statistics in Medicine*.

[17] Spiegelhalter A. T. D., Best N., Lunn D. (2014). *OpenBUGS User Manual*.

[18] Limmathurotsakul D., Jamsen K., Arayawichanont A. (2010). Defining the true sensitivity of culture for the diagnosis of melioidosis using bayesian latent class models. *PLoS ONE*.

[19] Geurden T., Claerebout E., Vercruysse J., Berkvens D. (2004). Estimation of diagnostic test characteristics and prevalence of Giardia duodenalis in dairy calves in Belgium using a Bayesian approach. *International Journal for Parasitology*.

[20] Pennello G. A. (2011). Bayesian analysis of diagnostic test accuracy when disease state is unverified for some subjects. *Journal of Biopharmaceutical Statistics*.

[21] Evans R. B., Erlandson K. (2004). Robust Bayesian prediction of subject disease status and population prevalence using several similar diagnostic tests. *Statistics in Medicine*.

