# Supplementary material for: Comparison of Two Bayesian Methods in Evaluation of the Absence of the Gold Standard Diagnostic Tests
Source: Biomed Res Int. 2019 Aug 21;2019:1374748. doi: 10.1155/2019/1374748 (PMC6720053; doi:10.1155/2019/1374748)
Supplement: Supplementary file — Text S1: two scenarios of Conditional Covariance Bayesian model. [file 1374748.f1.docx]

**Two** **scenarios of the Conditional covariance Bayesian method**

**(1). Conditional independence** **Scenario for** **Conditional covariance Bayesian method**

$$\left[ P_{00}=P\left( {T_{1}}^{-}\cap{T_{2}}^{-} \right)\cdots P_{11}=P\left( {T_{1}}^{+}\cap{T_{2}}^{+} \right) \right]$$

$$P_{00}=p\left( 1-{se}_{1} \right)\left( 1-{se}_{2} \right)+\left( 1-p \right){sp}_{1}{sp}_{2}$$

$$P_{01}=p\left( 1-{se}_{1} \right){se}_{2}+\left( 1-p \right){sp}_{1}\left( 1-{sp}_{2} \right)$$

$$P_{10}=p\left( 1-{se}_{2} \right){se}_{1}+\left( 1-p \right)\left( 1-{sp}_{1} \right){sp}_{2}$$

$$P_{11}=p\left( {se}_{1}*{se}_{2} \right)+\left( 1-p \right)\left( 1-{sp}_{1} \right)\left( 1-{sp}_{2} \right)$$

**(2). Conditional dependence** **Scenario for Conditional covariance Bayesian method**

$$\left[ P_{00}=P\left( {T_{1}}^{-}\cap{T_{2}}^{-} \right)\cdots P_{11}=P\left( {T_{1}}^{+}\cap{T_{2}}^{+} \right) \right]$$

$$P_{00}=p\left[ \left( 1-{se}_{1} \right)\left( 1-{se}_{2} \right)+cov\left( D_{p} \right) \right]+\left( 1-p \right)\left[ {sp}_{1}{sp}_{2}+cov\left( D_{n} \right) \right]$$

$$P_{01}=p\left[ \left( 1-{se}_{1} \right){se}_{2}-cov\left( D_{p} \right) \right]+\left( 1-p \right)\left[ {sp}_{1}\left( 1-{sp}_{2} \right)-cov\left( D_{n} \right) \right]$$

$$P_{10}=p\left[ \left( 1-{se}_{2} \right){se}_{1}-cov\left( D_{p} \right) \right]+\left( 1-p \right)\left[ \left( 1-{sp}_{1} \right){sp}_{2}-cov\left( D_{n} \right) \right]$$

$$P_{11}=p\left[ \left( {se}_{1}*{se}_{2} \right)+cov\left( D_{p} \right) \right]+\left( 1-p \right)\left[ \left( 1-{sp}_{1} \right)\left( 1-{sp}_{2} \right)+cov\left( D_{n} \right) \right]$$
