# Supplementary material for: Comparison of Two Bayesian Methods in Evaluation of the Absence of the Gold Standard Diagnostic Tests
Source: Biomed Res Int. 2019 Aug 21;2019:1374748. doi: 10.1155/2019/1374748 (PMC6720053; doi:10.1155/2019/1374748)
Supplement: Supplementary file — Text S2: two scenarios of the Bayesian probabilistic constraint model. [file 1374748.f2.docx]

**Two scenarios of the Bayesian probabilistic constraint model**

**(1). Conditional independence** **Scenario for Bayesian probabilistic constraint model**

**a. *Conditional probabilities***

Prevalence $P(D^{+})$ $[\theta_{1}]$

Se_1_ $P({T_{1}}^{+}|D^{+})$ $[\theta_{2}]$

Sp_1_  $P({T_{1}}^{-}|D^{-})$ $[\theta_{3}]$

$P({T_{2}}^{+}|D^{+}\cap{T_{1}}^{+})$ $[\theta_{4}]$

$P({T_{2}}^{+}|D^{+}\cap{T_{1}}^{-})$ $[\theta_{5}]$

$P({T_{2}}^{-}|D^{-}\cap{T_{1}}^{-})$ $[\theta_{6}]$

$P({T_{2}}^{-}|D^{-}\cap{T_{1}}^{+})$ $[\theta_{7}]$

**b. *Parameters***

p=$\theta_{1}$

Se_1=_$\theta_{2}$

Sp_1=_$\theta_{3}$

Se_2=_$\theta_{4}|\theta_{5}$

Sp_2=_$\theta_{6}|\theta_{7}$

**c. *Test result probabilities***

$$\left[ P\left( 00 \right)=P\left( {T_{1}}^{-}\cap{T_{2}}^{-} \right)\ldots P\left( 11 \right)=P\left( {T_{1}}^{+}\cap{T_{2}}^{+} \right) \right]$$

$$P\left( 00 \right)=\theta_{1}\left( 1-\theta_{2} \right)\left( 1-\theta_{5} \right)+\left( 1-\theta_{1} \right)\theta_{3}\theta_{6}=p\left( 1-{se}_{1} \right)\left( 1-{se}_{2} \right)+\left( 1-p \right){sp}_{1}{sp}_{2}$$

$$P\left( 01 \right)=\theta_{1}\left( 1-\theta_{2} \right)\theta_{5}+\left( 1-\theta_{1} \right)\theta_{3}\left( 1-\theta_{6} \right)=p\left( 1-{se}_{1} \right){se}_{2}+\left( 1-p \right){sp}_{1}\left( 1-{sp}_{2} \right)$$

$$P\left( 10 \right)=\theta_{1}\theta_{2}\left( 1-\theta_{4} \right)+\left( 1-\theta_{1} \right)\left( 1-\theta_{3} \right)\theta_{7}=p\left( 1-{se}_{2} \right){se}_{1}+\left( 1-p \right)\left( 1-{sp}_{1} \right){sp}_{2}$$

$$P\left( 11 \right)=\theta_{1}\theta_{2}\theta_{4}+\left( 1-\theta_{1} \right)\left( 1-\theta_{3} \right)\left( 1-\theta_{7} \right)=p\left( {se}_{1}*{se}_{2} \right)+\left( 1-p \right)\left( 1-{sp}_{1} \right)\left( 1-{sp}_{2} \right)$$

**(2). Conditional dependence** **Scenario for Bayesian probabilistic constraint model**

***a. Conditional probabilities***

Prevalence $P(D^{+})$ $[\theta_{1}]$

Se_1_ $P({T_{1}}^{+}|D^{+})$ $[\theta_{2}]$

Sp_1_  $P({T_{1}}^{-}|D^{-})$ $[\theta_{3}]$

$P({T_{2}}^{+}|D^{+}\cap{T_{1}}^{+})$ $[\theta_{4}]$

$P({T_{2}}^{+}|D^{+}\cap{T_{1}}^{-})$ $[\theta_{5}]$

$P({T_{2}}^{-}|D^{-}\cap{T_{1}}^{-})$ $[\theta_{6}]$

$P({T_{2}}^{-}|D^{-}\cap{T_{1}}^{+})$ $[\theta_{7}]$

***b. Parameters***

p=$\theta_{1}$

Se_1=_$\theta_{2}$

Sp_1=_$\theta_{3}$

Se_2=_$\theta_{2}\theta_{4}+\left( 1-\theta_{2} \right)\theta_{5}$

Sp_2=_$\theta_{3}\theta_{6}+\left( 1-\theta_{3} \right)\theta_{7}$

***c. Test result probabilities***

$$\left[ P\left( 00 \right)=P\left( {T_{1}}^{-}\cap{T_{2}}^{-} \right)\ldots P\left( 11 \right)=P\left( {T_{1}}^{+}\cap{T_{2}}^{+} \right) \right]$$

$$P\left( 00 \right)=\theta_{1}\left( 1-\theta_{2} \right)\left( 1-\theta_{5} \right)+\left( 1-\theta_{1} \right)\theta_{3}\theta_{6}$$

$$P\left( 01 \right)=\theta_{1}\left( 1-\theta_{2} \right)\theta_{5}+\left( 1-\theta_{1} \right)\theta_{3}\left( 1-\theta_{6} \right)$$

$$P\left( 10 \right)=\theta_{1}\theta_{2}\left( 1-\theta_{4} \right)+\left( 1-\theta_{1} \right)\left( 1-\theta_{3} \right)\theta_{7}$$

$$P\left( 11 \right)=\theta_{1}\theta_{2}\theta_{4}+\left( 1-\theta_{1} \right)\left( 1-\theta_{3} \right)\left( 1-\theta_{7} \right)$$
