# Supplementary material for: Comparison of Two Bayesian Methods in Evaluation of the Absence of the Gold Standard Diagnostic Tests
Source: Biomed Res Int. 2019 Aug 21;2019:1374748. doi: 10.1155/2019/1374748 (PMC6720053; doi:10.1155/2019/1374748)
Supplement: Supplementary file — Text S3: OpenBugs code. [file 1374748.f3.docx]

1. **OpenBugs Code for Model NC(Conditional Covariance Bayesian model without Prior) in conditional independence situation**

model{

#Likelihood

x[1:4]~dmulti(p[1:4],n)

p[1]<-pai*(1-se1)*(1-se2)+(1-pai)*sp1*sp2

p[2]<-pai*(1-se1)*se2+(1-pai)*sp1*(1-sp2)

p[3]<-pai*se1*(1-se2)+(1-pai)*(1-sp1)*sp2

p[4]<-pai*se1*se2+(1-pai)*(1-sp1)*(1-sp2)

#Prior

pai~dbeta(1,1)

se1~dbeta(1,1)

sp1~dbeta(1,1)

se2~dbeta(1,1)

sp2~dbeta(1,1)

}

#Data

list(x= c(130, 81, 191, 235), n = 637)

#Initial value

list(pai = 0.50, se1= 0.63, sp1 = 0.37, se2 = 0.49, sp2 = 0.51)

list(pai = 0.50, se1= 0.63, sp1 = 0.37, se2 = 0.49, sp2 = 0.51)

1. **OpenBugs Code for Model PC****(Conditional Covariance Bayesian model with Prior) in conditional independence situation**

model{

#Likelihood

x[1:4]~dmulti(p[1:4],n)

p[1]<-pai*(1-se1)*(1-se2)+(1-pai)*sp1*sp2

p[2]<-pai*(1-se1)*se2+(1-pai)*sp1*(1-sp2)

p[3]<-pai*se1*(1-se2)+(1-pai)*(1-sp1)*sp2

p[4]<-pai*se1*se2+(1-pai)*(1-sp1)*(1-sp2)

#Prior

pai ~ dbeta(3.991,5.579)

se1 ~ dbeta(41.77,5.005)

sp1 ~ dbeta(10.082,1.807)

se2 ~ dbeta(7.207,5.393)

sp2 ~ dbeta(20.067,7.920)

}

#end BUGS model specification

#Data

list(x= c(130, 81, 191, 235), n = 637)

#Initial value

list(pai = 0.50, se1= 0.63, sp1 = 0.37, se2 = 0.49, sp2 = 0.51)

list(pai = 0.50, se1= 0.63, sp1 = 0.37, se2 = 0.49, sp2 = 0.51)

1. **OpenBugs Code for Model NP(Bayesian probabilistic constraint model without Prior) in conditional independence situation**

#BUGS model specification begains here

model{

#Likelihood

x[1:4]~dmulti(p[1:4],N)

p[1]<-pai*(1-se1)*(1-se2)+(1-pai)*sp1*sp2

p[2]<-pai*(1-se1)*se2+(1-pai)*sp1*(1-sp2)

p[3]<-pai*se1*(1-se2)+(1-pai)*(1-sp1)*sp2

p[4]<-pai*se1*se2+(1-pai)*(1-sp1)*(1-sp2)

#Prior

pai~dbeta(1,1)

se1~dbeta(1,1)

sp1~dbeta(1,1)

se2~dbeta(1,1)

sp2~dbeta(1,1)

}

#end BUGS model specification

#Data

list(x= c(130, 81, 191, 235), n = 637)

#Initial value

list(pai = 0.49, se1= 0.61, sp1 = 0.37, se2 = 0.46, sp2 = 0.52)

list(pai = 0.49, se1= 0.61, sp1 = 0.37, se2 = 0.46, sp2 = 0.52)

1. **OpenBugs Code for Model PP(Bayesian probabilistic constraint model with Prior) in conditional independence situation**

#BUGS model specification begains here

model{

#Likelihood

x[1:4]~dmulti(p[1:4],N)

p[1]<-pai*(1-se1)*(1-se2)+(1-pai)*sp1*sp2

p[2]<-pai*(1-se1)*se2+(1-pai)*sp1*(1-sp2)

p[3]<-pai*se1*(1-se2)+(1-pai)*(1-sp1)*sp2

p[4]<-pai*se1*se2+(1-pai)*(1-sp1)*(1-sp2)

#Prior

pai~dbeta(75.83,66.73)

se1~dbeta(70.84,35.86)

sp1~dbeta(21.90,16.21)

se2~dbeta(99.74,56.18)

sp2~dbeta(112.32,130.69)

}

#end BUGS model specification#end BUGS model specification

#Data

list(x= c(130, 81, 191, 235), n = 637)

#Initial value

list(pai = 0.49, se1= 0.61, sp1 = 0.37, se2 = 0.46, sp2 = 0.52)

list(pai = 0.49, se1= 0.61, sp1 = 0.37, se2 = 0.46, sp2 = 0.52)

1. **OpenBugs Code for Model NC(Conditional Covariance Bayesian model without Prior) in conditional dependence situation**

#BUGS model specification begains here

model{

#Likelihood

x[1:4] ~ dmulti(p[1:4], n)

p[1] <- pi*((1-Se1)*(1-Se2)+covDp) + (1-pi)*(Sp1*Sp2+covDn)

p[2] <- pi*((1-Se1)*Se2-covDp) + (1-pi)*(Sp1*(1-Sp2)-covDn)

p[3] <- pi*(Se1*(1-Se2)-covDp) + (1-pi)*((1-Sp1)*Sp2-covDn)

p[4] <- pi*(Se1*Se2+covDp) + (1-pi)*((1-Sp1)*(1-Sp2)+covDn)

ls <- 0

us <- min(Se1,Se2) - Se1*Se2

lc <- 0

uc <- min(Sp1,Sp2) - Sp1*Sp2

covDn ~ dunif(lc, uc)

covDp ~ dunif(ls, us)

rhoD <- covDp / sqrt(Se1*(1-Se1)*Se2*(1-Se2))

rhoDc <- covDn / sqrt(Sp1*(1-Sp1)*Sp2*(1-Sp2))

#Prior

pi ~ dbeta(1,1) ### Mode=0.00005, 95% sure > 0.0001

Se1 ~ dbeta(1,1) ### Mode=0.9778, 95% sure > 0.90

Sp1 ~ dbeta(1,1) ### Mode=0.9681, 95% sure > 0.91

Se2 ~ dbeta(1,1) ### Mode=0.9778, 95% sure > 0.90

Sp2 ~ dbeta(1,1) ### Mode=0.9681, 95% sure > 0.91

}

#end BUGS model specification

#Data

list(x= c(130, 81, 191, 235), n = 637)

#Initial value

list(pi=0.51,Se1=0.63, Sp1=0.37, Se2=0.49, Sp2=0.51)

list(pi=0.51,Se1=0.63, Sp1=0.37, Se2=0.49, Sp2=0.51)

1. **OpenBugs Code for Model PC(Conditional Covariance Bayesian model with Prior) in conditional dependence situation**

#BUGS model specification begains here

model{

#Likelihood

x[1:4] ~ dmulti(p[1:4], n)

p[1] <- pi*((1-Se1)*(1-Se2)+covDp) + (1-pi)*(Sp1*Sp2+covDn)

p[2] <- pi*((1-Se1)*Se2-covDp) + (1-pi)*(Sp1*(1-Sp2)-covDn)

p[3] <- pi*(Se1*(1-Se2)-covDp) + (1-pi)*((1-Sp1)*Sp2-covDn)

p[4] <- pi*(Se1*Se2+covDp) + (1-pi)*((1-Sp1)*(1-Sp2)+covDn)

ls <- 0

us <- min(Se1,Se2) - Se1*Se2

lc <- 0

uc <- min(Sp1,Sp2) - Sp1*Sp2

covDn ~ dunif(lc, uc)

covDp ~ dunif(ls, us)

rhoD <- covDp / sqrt(Se1*(1-Se1)*Se2*(1-Se2))

rhoDc <- covDn / sqrt(Sp1*(1-Sp1)*Sp2*(1-Sp2))

#Prior

pi ~ dbeta(3.991,5.579)

Se1 ~ dbeta(41.77,5.005)

Sp1 ~ dbeta(10.082,1.807)

Se2 ~ dbeta(7.207,5.393)

Sp2 ~ dbeta(20.067,7.920)

}

#end BUGS model specification

#Data

list(x= c(130, 81, 191, 235), n = 637)

#Initial value

list(pi=0.51,Se1=0.63, Sp1=0.37, Se2=0.49, Sp2=0.51)

list(pi=0.51,Se1=0.63, Sp1=0.37, Se2=0.49, Sp2=0.51)

1. **OpenBugs Code for Model NP(Bayesian probabilistic constraint model without Prior) in conditional dependence situation**

#BUGS model specification begains here

model{

#Likelihood

x[1:4]~dmulti(p[1:4],N)

p[1]<-theta1*(1-theta2)*(1-theta5)+(1-theta1)*theta3*theta6

p[2]<-theta1*(1-theta2)*theta5+(1-theta1)*theta3*(1-theta6)

p[3]<-theta1*theta2*(1-theta4)+(1-theta1)*(1-theta3)*theta7

p[4]<-theta1*theta2*theta4+(1-theta1)*(1-theta3)*(1-theta7)

pai<-theta1

se1<-theta2

sp1<-theta3

se2<-theta2*theta4+(1-theta2)*theta5

sp2<-theta3*theta6+(1-theta3)*theta7

#Prior

theta1~dbeta(1,1)

theta2~dbeta(1,1)

theta3~dbeta(1,1)

theta4~dbeta(1,1)

theta5~dbeta(1,1)

theta6~dbeta(1,1)

theta7~dbeta(1,1)

}

#end BUGS model specification

#Data

list(x = c(130, 81, 191, 235), N = 637)

#Initial value

list(theta1 = 0.51, theta2 = 0.65, theta3 = 0.37, theta4 = 0.278, theta5 = 0.22, theta6 = 0.78, theta7 = 0.69)

list(theta1 = 0.5108, theta2 = 0.650, theta3 = 0.34, theta4 = 0.285, theta5 = 0.20, theta6 = 0.82, theta7 = 0.70)

1. **OpenBugs Code for Model PP(Bayesian probabilistic constraint model with Prior) in conditional dependence situation**

#BUGS model specification begains here

model{

#Likelihood

x[1:4]~dmulti(p[1:4],N)

p[1]<-theta1*(1-theta2)*(1-theta5)+(1-theta1)*theta3*theta6

p[2]<-theta1*(1-theta2)*theta5+(1-theta1)*theta3*(1-theta6)

p[3]<-theta1*theta2*(1-theta4)+(1-theta1)*(1-theta3)*theta7

p[4]<-theta1*theta2*theta4+(1-theta1)*(1-theta3)*(1-theta7)

pai<-theta1

se1<-theta2

sp1<-theta3

se2<-theta2*theta4+(1-theta2)*theta5

sp2<-theta3*theta6+(1-theta3)*theta7

#Prior

theta1~dbeta(75.83,66.73)

theta2~dbeta(70.84,35.86)

theta3~dbeta(21.90,16.21)

theta4~dbeta(99.74,56.18)

theta5~dbeta(53.43,100.05)

theta6~dbeta(143.25,85.38)

theta7~dbeta(112.32,130.69)

}

#end BUGS model specification

#Data

list(x = c(130, 81, 191, 235), N = 637)

#Initial value

list(theta1 = 0.51, theta2 = 0.65, theta3 = 0.37, theta4 = 0.278, theta5 = 0.22, theta6 = 0.78, theta7 = 0.69)

list(theta1 = 0.5108, theta2 = 0.650, theta3 = 0.34, theta4 = 0.285, theta5 = 0.20, theta6 = 0.82, theta7 = 0.70)
